# Optimization of immune receptor-related hypersensitive cell death response assay using agrobacterium-mediated transient expression in tobacco plants

**DOI:** 10.1186/s13007-022-00893-z

**Published:** 2022-05-02

**Authors:** Sung Un Huh

**Affiliations:** Department of Biological Science, Kunsan National University, Gunsan, 54150 Republic of Korea

**Keywords:** NLR-type immune receptor, Pathogen effector, Cell death, Paired NLR, Tobacco, Agrobacterium, Hypersensitive response

## Abstract

**Background:**

The study of the regulatory mechanisms of evolutionarily conserved Nucleotide-binding leucine-rich repeat (NLR) resistance (R) proteins in animals and plants is of increasing importance due to understanding basic immunity and the value of various crop engineering applications of NLR immune receptors. The importance of temperature is also emerging when applying NLR to crops responding to global climate change. In particular, studies of pathogen effector recognition and autoimmune activity of NLRs in plants can quickly and easily determine their function in tobacco using agro-mediated transient assay. However, there are conditions that should not be overlooked in these cell death-related assays in tobacco.

**Results:**

Environmental conditions play an important role in the immune response of plants. The system used in this study was to establish conditions for optimal hypertensive response (HR) cell death analysis by using the paired NLR RPS4/RRS1 autoimmune and AvrRps4 effector recognition system. The most suitable greenhouse temperature for growing plants was fixed at 22 °C. In this study, RPS4/RRS1-mediated autoimmune activity, RPS4 TIR domain-dependent cell death, and RPS4/RRS1-mediated HR cell death upon AvrRps4 perception significantly inhibited under conditions of 65% humidity. The HR is strongly activated when the humidity is below 10%. Besides, the leaf position of tobacco is important for HR cell death. Position #4 of the leaf from the top in 4–5 weeks old tobacco plants showed the most effective HR cell death.

**Conclusions:**

As whole genome sequencing (WGS) or resistance gene enrichment sequencing (RenSeq) of various crops continues, different types of NLRs and their functions will be studied. At this time, if we optimize the conditions for evaluating NLR-mediated HR cell death, it will help to more accurately identify the function of NLRs. In addition, it will be possible to contribute to crop development in response to global climate change through NLR engineering.

## Introduction

Recent climate change is changing the map of crop production. An increase in temperature can aggravate crop damage caused by pathogens [1, 2]. To actively respond to these rapid changes, a rapid response through genetic engineering is required.

Plant pathogens inject effector proteins by manipulating cellular processes via type three secreted systems (T3SEs) into host cells [3]. Nucleotide-binding leucine-rich repeat receptor (NLR) proteins recognize specific effectors and trigger effector-triggered immunity (ETI), which is often associated with localized programmed cell death, known as the hypersensitive response (HR) and limits pathogen proliferation [4]. Plant NLRs are structurally and evolutionarily similar to animal NLRs [5]. How plant NLRs signal immune responses remains largely unknown when compared to similar animal systems.

Like animals, paired NLRs exist in plants, and their functions are divided into sensor NLR and helper NLR. In particular, sensor NLRs have a role in recognizing effectors via the integrated domain and inhibit the autoimmune activity of helper NLRs [6]. For example, this is the case with RPS4/RRS1. RRS1 is known to recognize effectors secreted by three different pathogens [7, 8]. Interestingly, artificially extending the N- or C-terminus of RRS1 activates RPS4 autoimmunity without effector recognition [9]. That is, an autoimmune system and an effector recognition system using RPS4/RRS1 paired NLR can be studied together.

*Agrobacterium*-mediated transient analysis is widely used to evaluate the function of NLRs. This is because the clear phenotype of HR cell death can be assessed with various combinations of NLRs and effectors in a relatively short time without obtaining the transgenic plants. It is known that NLR-mediated cell death is influenced by environmental factors such as temperature and humidity [10, 11]. In particular, the immunity of plants decreases with increasing temperature conditions and affects effector recognition of NLRs [12]. If so, it is important to clarify the conditions for an effective HR cell death assay using an *agrobacterium*-mediated transient system in tobacco plants.

In this study, we confirmed that humidity and plant leaf position, excluding temperature conditions, were important for effectively confirming HR cell death assays in tobacco plants. The RPS4/RRS1-mediated cell death was activated by AvrRps4 perception in tobacco plants; this HR cell death was suppressed at 65% humidity. However, at 10% humidity, HR cell death was enhanced. Additionally, the infiltrated-leaf position of tobacco is important. Position #4 of recent leaves from the tops in 4–5 weeks old plants showed the most effective HR cell death. Under these distinct conditions, performing NLR-mediated HR upon effector recognition or autoimmune activation may yield more accurate results.

## Results

### The NLR RPS4/RRS1 pair can be used in the cell death assay system, which is divided into autoimmune activity and effector-triggered hypersensitive cell death response

**Fig. 1:**
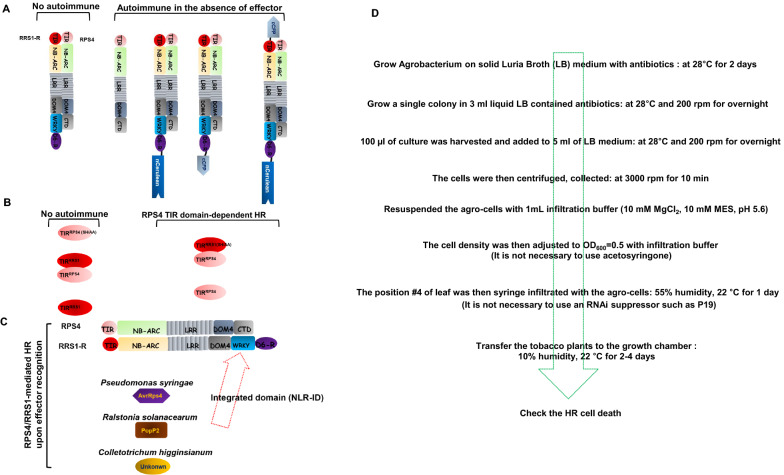
Three HR cell death systems utilizing the paired NLR RPS4/RRS1. **A** The helper RPS4 autoimmune activity is suppressed by sensor RRS1. RPS4 exhibits autoimmune activity. RRS1-R-cCFP, RRS1-R-nCerulean, and cCFP-RRS1-R-nCerulean trigger autoimmune with RPS4. **B** The TIR domain of helper RPS4 is capable of generating an autoimmune signal by itself. TIR^RPS4^-dependent HR can be repressed by coexpressing of TIR domain of RRS1. **C** RPS4/RRS1 directly recognizes effectors and induces NLR-mediated HR cell death. **D** Schematic of *Agrobacterium*-mediated transient expression for HR cell death assay

Two NLR immune receptor proteins encoded by genetically linked genes function together as paired NLRs. They are divided into sensor NLR and helper NLR according to their function. Sensor NLR usually has a non-canonical domain called the integrated domain (ID) which can recognize cognate effector proteins [13, 14]. Overexpression of helper NLR caused HR-like cell death in the absence of sensor NLR. If coexpressed sensor/helper NLRs, the HR cell death is inhibited (Fig. 1A) [13, 15]. When the N-terminus or C-terminus of sensor NLR RRS1 was extended, RPS4-dependent autoimmunity appeared without effector perception (Fig. 1A). This is probably because the protein conformational change of RRS1 loses regulation of RPS4 autoimmune activity [9]. In the engineering of sensor NLRs, it must be careful when engineering to employ specific proteins. TIR (Toll-like, Interleukin-1 receptor, Resistance protein) or CC (coiled-coil) of the helper NLR act as a signaling domain. The TIR^RPS4^ domain alone can activate TIR-dependent autoimmune responses and TIR^RRS1^ can suppress this HR cell death [16]. TIR domain mutant RPS4(SH/AA) fail to activate HR, suggesting that TIR^RPS4^ oligomerization is required for generating the HR signal (Fig. 1B). RRS1-R from accessions Ws-2 forms an immune receptor complex with RPS4 that recognizes *Pseudomonas syringae* AvrRps4, *Ralstonia solanacearum* PopP2, and unknown effectors [7, 8, 17, 18]. Thus, the paired RPS4/RRS1-R can prevent infection by three distinct pathogens and it can be used as a very useful tool for crop engineering (Fig. 1C). We summarize the following experimental procedures based on the RPS4/RRS1 system capable of performing these three types of HR cell death assays using the *Agrobacterium*-mediated transient expression in tobacco plant (Fig. 1D).

### Both RRS1-S and RRS1-R fused with fluorescent proteins under low humidity conditions exhibited RPS4-dependent HR cell death

**Fig. 2:**
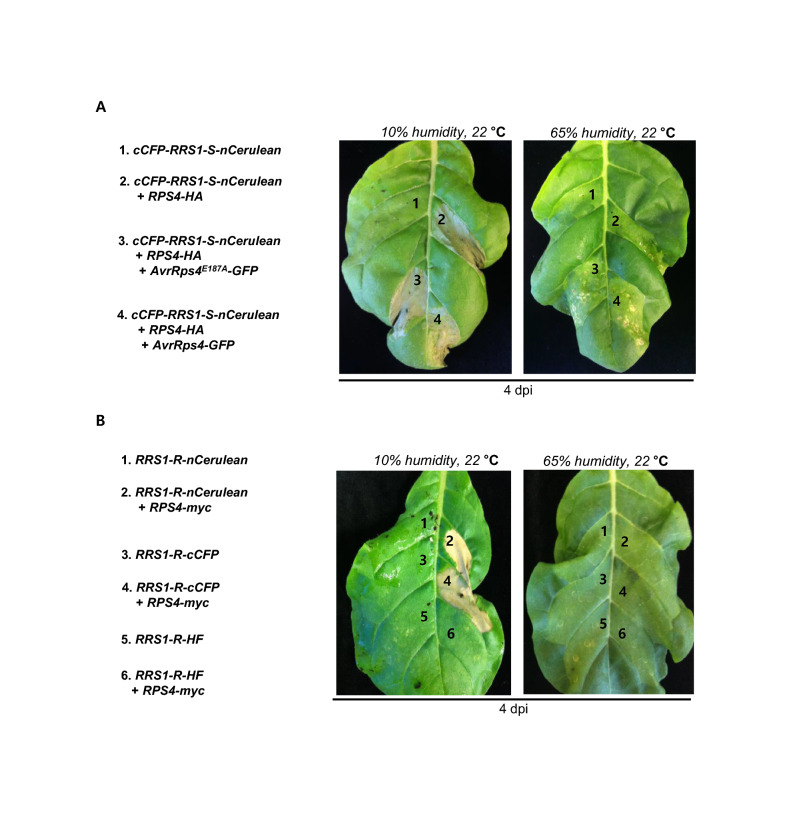
RPS4/RRS1 autoimmune activity is enhanced by low humidity. **A** cCFP-RRS1S-nCerulean triggered RPS4-depentent autoimmune cell death in *N. tabacum*. *35S::cCFP-RRS1-S-nCerulean* or *35S::cCFP-RRS1-S-nCerulean*/*35S::RPS4-HA or 35S::cCFP-RRS1-S-nCerulean*/*35S::RPS4-HA*/*35S::AvrRps4-GFP* or *35S::cCFP-RRS1-S-nCerulean*/*35S::RPS4-HA*/*35S::AvrRps4*^*E187A*^*-GFP* were transiently co-expressed in *N. tabacum* leaves. HR cell death was confirmed on the 4 days. **B** RRS1-R-nCerulean and RRS1-R-nCFP triggered RPS4-depentent autoimmune cell death in *N. tabacum*. *35S::RRS1-R-nCerulean* or *35S::RRS1-R-nCerulean*/*35S::RPS4-Myc* or *35S::RRS1-R-cCFP* or *35S::RRS1-R-cCFP*/*35S::RPS4-Myc* or *35S::RRS1-R-HF or 35S::RRS1-R-HF/35S::RPS4-Myc* were transiently co-expressed in *N. tabacum* leaves. HR cell death was confirmed on the 4 days

Sensor NLR is a good material for developing crops that recognize various pathogens by introducing new IDs. The positioning may alter the overall sensor NLR protein size or fuse new IDs into the N- or C-terminus [19]. Bimolecular fluorescence complementation (BiFC) methods to confirm intra-/intercellular interactions should evaluate their function when applied to sensor NLRs [9, 20]. We found that overexpression of *cCFP-RRS1-S-nCerulean* activated RPS4-depenent HR cell death in low humidity conditions, but this cell death was suppressed in the high humidity condition (Fig. 2A). At 4 dpi, we could not detect any difference between *cCFP-RRS1-S-nCerulean/RPS4-HA* mediated autoimmune activity and *cCFP-RRS1-S-nCerulean/RPS4-HA* mediated HR cell death upon AvrRps4 perception (Fig. 2A). As a result, it was confirmed that the humidity condition at a temperature of 22 °C affected HR cell death.

In the case of RRS1-R, unlike RRS1-S, 83 amino acids are extended at the C-terminus, which is known to play a decisive role in recognizing PopP2 [21]. We tested changes in autoimmunity under different humidity conditions using RRS1R-cCFP and RRS1-R-nCerulean. Consistent with *cCFP-RRS1-S-nCerulean*, both *RRS1-R-cCFP* and *RRS1-R-nCerulean* activated RPS4-dependent HR cell death under low humidity. High humidity can inhibit HR cell death in *N. tabacum* (Fig. 2B). Thus, humidity is an important determinant of HR cell death activation by paired NLR autoimmune.

### Agro-infiltrated leaf location in the tobacco plant is also a critical factor in HR cell death assays

**Fig. 3:**
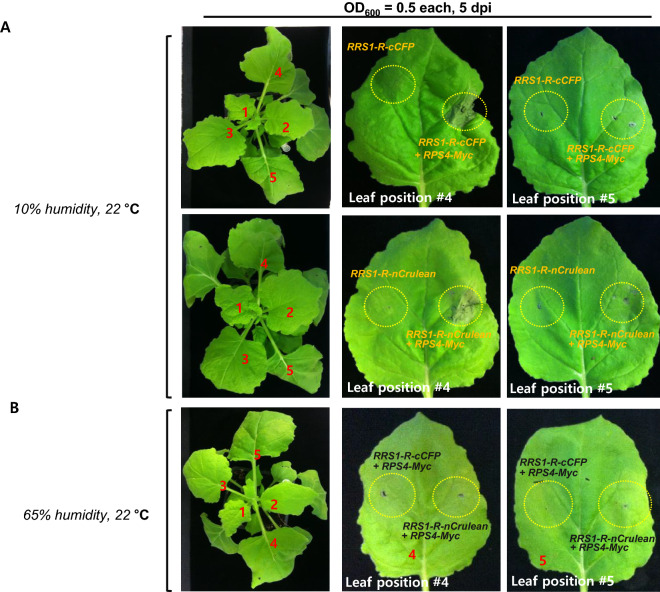
Effect of leaf position and humidity on RPS4/RRS1-mediated autoimmunity in *N. benthamiana. A* The autoimmune activity of RPS4-mediated RRS1-cCFP or RRS1-nCerulean at leaf positions #4 and #5 was different at low humidity. The positions of the leaves are numbered from top to bottom. *35S::RRS1-R-cCFP* or *35S::RRS1-R-cCFP*/*35S::RPS4-Myc or 35S::RRS1-R-nCerulean* or *35S::RRS1-R-nCerulean*/*35S::RPS4-Myc* were transiently co-expressed in *N. benthamiana* leaf positions #4 and #5. The HR cell death was confirmed on the 5 days. **B** The autoimmune activity of RPS4-mediated RRS1-cCFP or RRS1-nCerulean at leaf positions #4 and #5 was suppressed by high humidity. *35S::RRS1-R-cCFP*/*35S::RPS4-Myc* or *35S::RRS1-R-nCerulean*/*35S::RPS4-Myc* were transiently co-expressed in *N. benthamiana* leaf positions #4 and #5. The HR cell death was confirmed on the 5 days

HR cell death assays using tobacco plants often show differences in the intensities of HR cell death. In particular, if agro-infiltration is performed using various leaves in one plant, the same cell death cannot be obtained. To confirm that these differences occurred, leaf positions of 4–5 week old *N. benthamiana* were numbered from top to bottom. In leaf position #4, coexpression of *RRS1-R-cCFP/RPS4-Myc or RRS1-R-nCerulean*/*RPS4-Myc* exhibited strong HR cell death but not leaf position #5 (Fig. 3A). Consistently, this autoimmune activity was suppressed by high humidity in *N. benthamiana* (Fig. 3B). These results suggested that HR cell death is also affected by infiltrated leaf position and can be more easily detected under a relatively low humidity condition.

### Agro-infiltrating leaf position is an important factor to evaluate HR cell death in both TIR^RPS4^-mediated cell death and RPS4/RRS1-mediated effector-triggered cell death

NLRs are divided into two groups depending on the type of N-terminal domain. The N-terminal coiled-coil (CC) domain is called CNLs (CC-NLRs), and those with the N-terminal Toll/interleukin-1 receptor (TIR) domain is called TNLs (TIR-NLRs) [22]. Evolutionally, bacterial TIR domain proteins have NADase enzymatic activity that generates a non-canonical variant cyclic ADPR (cADPR) molecule and cleaves NAD^+^ (nicotinamide adenine dinucleotide) [23]. The TIR domain of plant NLR also has NADase activity and is required downstream signaling [24, 25]. The overexpression of the TIR^RPS4^ domain is sufficient to activate autoimmune responses, and oligomerization of the TIR^RPS4^ domain is required for plant immune signaling [16]. In TIR-mediated immunity studies, the importance of TIR function evaluation is increasing.

**Fig. 4:**
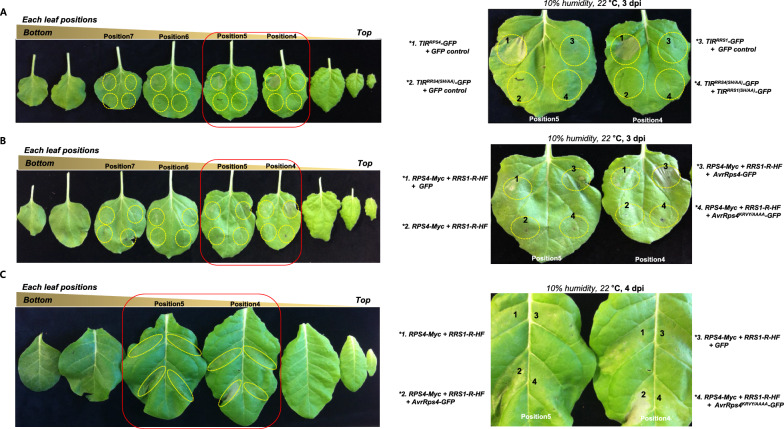
Effect of leaf position on TIR^RPS4^-mediated autoimmune and RPS4/RRS1-mediated effector triggered HR cell death in tobacco plants. **A** TIR^RPS4^-dependent autoimmune activity is different for each leaf position. *N. benthamiana* leaf was transiently agro-infiltrated with *35S::TIR*^*RPS4*^*-GFP*/*35S::GFP* or inactive mutant *35S::TIR*^*RPS4(SH/AA)*^*-GFP*/*35S::GFP* or *35S::TIR*^*RRS1*^*-GFP*/*35S::GFP* or *35S::TIR*^*RPS4(SH/AA)*^-*GFP*/inactive mutant *35S::TIR*^*RRS1(SH/AA)*^*-GFP* at leaf positions #4-#7. The HR cell death was confirmed on the 3 days. **B** RPS4/RRS1-mediated effector triggered HR cell death exhibited differently infiltrated leaf position in *N. benthamiana*. Agro-infiltration was performed with transiently coexpressed *35S::RRS1-R-HF*/*35S::RPS4-Myc with 35S::GFP or 35S::AvrRps4-GFP or* non-functional mutant *35S::AvrRps4*^*KRVY/AAAA*^*-GFP* at leaf positions #4-#7 in *N. benthamiana*. The HR cell death for leaf positions #4-#5 is enlarged. **C** RPS4/RRS1-mediated effector triggered HR cell death exhibited differently infiltrated leaf position in *N. tabacum*. Agro-infiltration was performed with transiently coexpressed *35S::RRS1-R-HF*/*35S::RPS4-Myc with 35S::GFP or 35S::AvrRps4-GFP or* non-functional mutant *35S::AvrRps4*^*KRVY/AAAA*^*-GFP* at leaf positions #4-#5 in *N. tabacum*. HR cell death for leaf positions #4-#5 is enlarged. The HR cell death was confirmed on the 4 days

*N. benthamiana* is an excellent system for studying cell death induced by the TIR/CC domains. TIR^RPS4^ was used to find suitable leaf positions for HR cell death assay in *N. benthamiana*. When co-delivered *35S::TIR*^*RPS4*^*-GFP*/*35S::GFP* control in leaf positions #4-#7, the HR cell death is detected in leaf position #4 and #5 (Fig. 4A). In leaf position #4, TIR^RPS4^-mediated autoimmune cell death was more potent than in leaf position #5 (Fig. 4A). In leaf positions #6 and #7, HR cell death was not detected (Fig. 4A). If co-expressed *35S::TIR*^*RPS4(SH/AA)*^*-GFP*/*35S::GFP* control, It cannot induce HR cell death because of inhibition of TIR^RPS4^ oligomerization (Fig. 4A).

It is also checked whether RPS4/RRS1 is functional in *N. benthamiana* system. When co-delivered *35S::RRS1-R-HF*/*35S::RPS4-Myc*/*35S::AvrRps4-GFP,* clear HR cell death was observed in the leaf positions #4, but weak HR cell death was exhibited in the leaf positions #5 (Fig. 4B). However, RPS4/RRS1-mediated HR cell death upon AvrRps4 perception is not observed in leaf positions #6-#7 (Fig. 4B). To determine whether HR intensity was determined by leaf position in *N. tabacum* as in *N. benthamiana*. Similarly, a combination of *35S::RRS1-R-HF*/*35S::RPS4-Myc*/*35S::AvrRps4-GFP* was co-expressed at leaf positions #4-#5 in *N. tabacum*. HR cell death phenotype was checked at 4 dpi. As expected, HR cell death was stronger in leaf position #4 than #5 (Fig. 4C). In effector-triggered HR cell death, the position of the leaf used for agro-infiltration affects the intensity of HR cell death, in both *N. benthamiana* and *N. tabacum*.

**Fig. 5:**
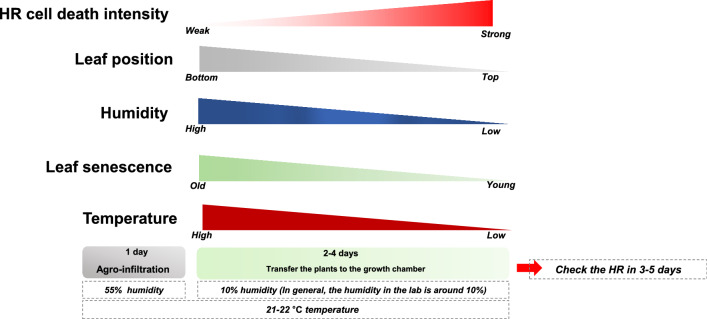
NLR-mediated HR cell death intensity depends on leaf position and humidity. Schematic showing the intensity of HR cell death according to temperature, humidity, and leaf position based on the results

Based on the results, we have summarized the important factors in the tobacco-based agro-infiltration cell death assay (Fig. 5). In most reported cases, high temperature suppressed HR as well as disease resistance. The high temperature suppressed plant resistance under various experimental conditions [10, 11]. In the HR cell death analysis, humidity and leaf position used for infiltration was the most important factors. Ultimately, it is expected to be of great help in experiments to confirm the cell death phenotype that exhibits various NLRs under these optimal conditions. In addition, when preparing a sample for co-immunoprecipitation (co-IP) or western blot analysis rather than a cell death assay, if the humidity is high and the sample is collected from a location other than location #4, sufficient experiments are possible.

## Discussion

In this study, we developed and optimized an agro-mediated transient cell death assay in tobacco plants. Tobacco plants are the most effective functional research system as various types of plant NLRs have been discovered through whole genome sequencing (WGS) and resistance gene enrichment sequencing (RenSeq) [26–31]. In particular, it is possible to quickly and easily identify which effector NLR recognizes or which domain regulates NLR autoimmune activity through phenotypes such as HR cell death. On the other hand, it is difficult to confirm the intracellular localization of the NLR fused with a fluorescent protein due to autoimmune activity. Some NLRs do not produce distinct HR responses in tobacco plants. It will be possible to determine the optimal HR cell death phenotype in NLR studies using low humidity conditions and infiltrated leaf positions.

The regulation mechanism of NLR-mediated humidity-sensitive HR is not fully understood. However, this is probably similar to temperature-sensitive HR cell death. Although there are exceptions and not much research has been done, high temperatures and humidity suppressed HR and affected plant immunity [10, 11, 32, 33]. For example, dwarf phenotype of autoimmune *snc1-1* is suppressed at 28 °C [34]. *Arabidopsis* U-box ubiquitin ligase SAUL1 regulated senescence and cell death. The *saul1-1* mutant showed autoimmune activity. An autoimmune phenotype of *saul1-1* was rescued by higher relative humidity and higher temperatures. The *saul1-1* phenotype regulated by EDS1/PAD4 dependent signaling pathway [32]. This implied that NLR-mediated cell death also might be connected with EDS1/PAD4 pathway. Salicylic acid (SA) may play an important role in the thermoregulation of plant NLR-mediated cell death [33].

Leaf senescence is one of the programmed cell death (PCD) and is regulated by the ethylene (ET) hormone [35]. Although SA regulates key pathways of plant cell death and immunity, this regulation is inhibited by ET [33]. Interestingly, ET did not affect leaf senescence in young seedlings [36]. There is a sufficient possibility that newly generated upper leaves can escape the effects of ET when used for agro-infiltration. Arabidopsis onset of leaf death (*old*) mutants is allelic to *CONSTITUTIVE EXPRESSER OF PR GENE5* (*CPR5*), which showed senescence symptoms in young seedlings [37, 38]. Interestingly, CPR5 maintains the steady state level of nicotinamide adenine dinucleotide (NAD) [39], suggesting that NAD homeostasis also may affect NLR-mediated cell death including autoimmune activity. CPR5 associated with a novel nucleoporin PLANT NUCLEAR ENVELOPE TRANSMEMBRANE 1 (PNET1) [40]. Function of human PNET1 homologs is important to cell cycle regulation [41]. This suggests that CPR5-PNET1 may have a dual function between cell cycle and immune pathways in the nuclear pore. Thus, leaf position-dependent HR cell death might be associated with cell cycle and immunity.

## Conclusions

Optimized transient HR cell death assay conditions for NLR studies using tobacco plants are proposed. When temperature, humidity, and leaf location conditions are optimally adjusted, various NLR-mediated effectors can induce HR apoptosis and observe an autoimmune phenotype.

## Methods

### Plant materials and growth conditions

*Nicotiana tabacum* cv. Petite Gerard and *Nicotiana benthamiana* plants were sown on soil and grown at 22 °C under long day conditions (16 h light/8 h dark) with 55% relative humidity in growth room. For the HR assay, the plants were placed in the growth room for 24 h after infiltration, and then the plants were transferred to the growth chamber to maintain the humidity at 10% or 65%.

### *Agrobacterium* strains and vector constructions

*Agrobacterium tumefaciens* GV3101 was used in infiltration assays with tobacco leaves. The *Agrobacterium* strain GV3101 competent cells were thawed on ice and added 1 μL recombined plant expression vector, then kept on ice for 5 min. Then the mixture was fast frozen in liquid nitrogen for 5 min, followed by an incubation at 37 ℃ for 5 min. After that, the mixture was kept in ice for 5 min and added 1 mL fresh Luria Broth (LB) liquid medium. After a culture in shaker for 1 h at 28 ℃, 200 rpm, 100 μL cells were plated on an LB agar plate containing rifampicin (25 mg/L) and kanamycin (100 mg/L), and then cultured for 2 days at 28 ℃.

Briefly, genomic fragments of full-length RRS1-R and RRS1-S were PCR-amplified from *Arabidopsis thaliana* genomic DNA, accessions Ws-2 and Col-0. The genomic fragments of RRS1 were PCR-amplified with primers containing 4 bp specific overhangs and *Bsa*I recognition sequence and cloned into the pCR8/GW/TOPO (ThermoFisher). The resulting pCR8 constructs were then used for Golden Gate assembly in pICH86988 [42]. C-cCFP, C-nCerulean, and N-cCFP tags were introduced into RRS1 [21]. *35S::RPS4-HA*, *35S::RPS4-Myc*, *35S::RRS1-R-HF*, *35S::AvrRps4-GFP*, *35S::AvrRps4*^*E187A*^*-GFP*, *35S::AvrRps4*^*KRVY/AAAA*^*-GFP*, *35S::TIR*^*RPS4*^*-GFP*, *35S::TIR*^*RPS4(SH/AA)−*^*GFP*, and *35S::TIR*^*RRS1(SH/AA)−*^*GFP* used in this study were described previously [15–17, 21].

### *Agrobacterium tumefaciens* infiltration in tobacco plant

A single positive colony of *Agrobacterium* was inoculated in 3 mL LB liquid medium (25 mg/L rifampicin and 100 mg/L kanamycin) and cultured for 1 day in a shaker at 28 ℃, 200 rpm. Then, 100 μL *Agrobacterium* cells were transferred into 5 mL fresh LB liquid medium supplemented with the above-mentioned antibiotics and cultured overnight at 28 ℃, 200 rpm. The *agrobacterium* cells were pelleted by centrifugation (3000 rpm, 10 min) and resuspended with 1 mL infiltration buffer (10 mM MgCl_2_, 10 mM MES, pH 5.6). The re-suspended agro-cells were then diluted to OD_600_ = 0.5 with infiltration buffer. Fully expanded leaves of 4–5 weeks old tobacco plant were syringe-infiltrated in the abaxial region. The leaf-infiltrated plants were dried with paper, and the humidity in the greenhouse was maintained at 55%. After 1 day, the plants were transferred to growth chamber with a 16-h light/8-h dark photoperiod at 22 °C, 65% humidity or transferred to growth chamber at 22 °C, 10% humidity. It is possible to test in a laboratory with a relative humidity of 10% and at 20–22 °C. The HR phenotype was confirmed at 3–5 dpi.

## Data Availability

All data generated or analysis during this study is included in published article. The materials are available from the corresponding author on reasonable request.
