# Correction to: Optimization of immune receptor-related hypersensitive cell death response assay using agrobacterium-mediated transient expression in tobacco plants

**DOI:** 10.1186/s13007-022-00924-9

**Published:** 2022-07-18

**Authors:** Sung Un Huh

**Affiliations:** Department of Biological Science, Kunsan National University, Gunsan, 54150 Republic of Korea

## Correction to: Plant Methods (2022) 18:57 https://doi.org/10.1186/s13007-022-00893-z

In the original publication of the article the references in the Reference section were erroneously published. The corrected references were provided below. The original article [1] has been corrected.

References

1. Chaloner TM, Gurr SJ, Bebber DP. Plant pathogen infection risk tracks global crop yields under climate change. Nat Clim Change. 2021;11(8):710–15.
2. Velasquez AC, Castroverde CDM, He SY. Plant-pathogen warfare under changing climate conditions. Curr Biol. 2018;28(10):R619–34.
3. McCann HC, Guttman DS. Evolution of the type III secretion system and its effectors in plant–microbe interactions. New Phytol .2008;177(1):33–47.
4. Pruitt RN, Gust AA, Nurnberger T. Plant immunity unified. Nat Plants. 2021;7(4):382–83.
5. Bentham A, Burdett H, Anderson PA, Williams SJ, Kobe B. Animal NLRs provide structural insights into plant NLR function. Ann Bot. 2017;119(5):827–702.
6. Adachi H, Derevnina L, Kamoun S. NLR singletons, pairs, and networks: evolution, assembly, and regulation of the intracellular immunoreceptor circuitry of plants. Curr Opin Plant Biol. 2019;50:121–31.
7. Narusaka M, Shirasu K, Noutoshi Y, Kubo Y, Shiraishi T, Iwabuchi M, Narusaka Y. RRS1 and RPS4 provide a dual Resistance-gene system against fungal and bacterial pathogens. Plant J. 2009;60(2):218–26.
8. Birker D, Heidrich K, Takahara H, Narusaka M, Deslandes L, Narusaka Y, Reymond M, Parker JE, O'Connell R. A locus conferring resistance to *Colletotrichum higginsianum* is shared by four geographically distinct Arabidopsis accessions. Plant J. 2009, 60(4):602–13.
9. Guo H, Ahn HK, Sklenar J, Huang J, Ma Y, Ding P, Menke FLH, Jones JDG. Phosphorylation-regulated activation of the arabidopsis RRS1-R/RPS4 immune receptor complex reveals two distinct effector recognition mechanisms. Cell Host Microbe. 2020;27(5):769–781.e766.
10. Menna A, Nguyen D, Guttman DS, Desveaux D. Elevated temperature differentially influences effector-triggered immunity outputs in arabidopsis. Front Plant Sci. 2015;6:995.
11. Kiraly L, Hafez YM, Fodor J, Kiraly Z. Suppression of tobacco mosaic virus-induced hypersensitive-type necrotization in tobacco at high temperature is associated with downregulation of NADPH oxidase and superoxide and stimulation of dehydroascorbate reductase. J Gen Virol. 2008;89(Pt 3):799–808.
12. Desaint H, Aoun N, Deslandes L, Vailleau F, Roux F, Berthome R. Fight hard or die trying: when plants face pathogens under heat stress. New Phytol. 2021;229(2):712–34.
13. Cesari S, Kanzaki H, Fujiwara T, Bernoux M, Chalvon V, Kawano Y, Shimamoto K, Dodds P, Terauchi R, Kroj T. The NB-LRR proteins RGA4 and RGA5 interact functionally and physically to confer disease resistance. EMBO J. 2014;33(17):1941–59.
14. Maqbool A, Saitoh H, Franceschetti M, Stevenson CE, Uemura A, Kanzaki H, Kamoun S, Terauchi R, Banfield MJ. Structural basis of pathogen recognition by an integrated HMA domain in a plant NLR immune receptor. Elife. 2015;4:e08709.
15. Huh SU, Cevik V, Ding P, Duxbury Z, Ma Y, Tomlinson L, Sarris PF, Jones JDG: Protein–protein interactions in the RPS4/RRS1 immune receptor complex. PLoS Pathog. 2017;13(5):e1006376.
16. Williams SJ, Sohn KH, Wan L, Bernoux M, Sarris PF, Segonzac C, Ve T, Ma Y, Saucet SB, Ericsson DJ et al. Structural basis for assembly and function of a heterodimeric plant immune receptor. Science. 2014;344(6181):299–303.
17. Sohn KH, Hughes RK, Piquerez SJ, Jones JD, Banfield MJ. Distinct regions of the Pseudomonas syringae coiled-coil effector AvrRps4 are required for activation of immunity. Proc Natl Acad Sci U S A. 2012;109(40):16371–376.
18. Tasset C, Bernoux M, Jauneau A, Pouzet C, Briere C, Kieffer-Jacquinod S, Rivas S, Marco Y, Deslandes L. Autoacetylation of the *Ralstonia solanacearum* effector PopP2 targets a lysine residue essential for RRS1-R-mediated immunity in Arabidopsis. PLoS Pathog. 2010;6(11):e1001202.
19. Grund E, Tremousaygue D, Deslandes L. Plant NLRs with integrated domains: unity makes strength. Plant Physiol. 2019;179(4):1227–35.
20. Ma Y, Guo H, Hu L, Martinez PP, Moschou PN, Cevik V, Ding P, Duxbury Z, Sarris PF, Jones JDG. Distinct modes of derepression of an Arabidopsis immune receptor complex by two different bacterial effectors. Proc Natl Acad Sci U S A. 2018;115(41):10218–227.
21. Sarris PF, Duxbury Z, Huh SU, Ma Y, Segonzac C, Sklenar J, Derbyshire P, Cevik V, Rallapalli G, Saucet SB et al. A plant immune receptor detects pathogen effectors that target wrky transcription factors. Cell. 2015;161(5):1089–1100.
22. Maruta N, Burdett H, Lim BYJ, Hu X, Desa S, Manik MK, Kobe B: Structural basis of NLR activation and innate immune signalling in plants. Immunogenetics. 2022;74(1):5-26.
23. Essuman K, Summers DW, Sasaki Y, Mao X, Yim AKY, DiAntonio A, Milbrandt J. TIR domain proteins are an ancient family of NAD( +)-consuming enzymes. Curr Biol. 2018;28(3):421–30.e424.
24. Horsefield S, Burdett H, Zhang X, Manik MK, Shi Y, Chen J, Qi T, Gilley J, Lai JS, Rank MX et al. NAD( +) cleavage activity by animal and plant TIR domains in cell death pathways. Science. 2019;365(6455):793–799.
25. Wan L, Essuman K, Anderson RG, Sasaki Y, Monteiro F, Chung EH, Osborne Nishimura E, DiAntonio A, Milbrandt J, Dangl JL et al.TIR domains of plant immune receptors are NAD( +)-cleaving enzymes that promote cell death. Science. 2019;365(6455):799–803.
26. Li Q, Jiang XM, Shao ZQ. Genome-Wide Analysis of NLR disease resistance genes in an updated reference genome of barley. Front Genet. 2021;12:694682.
27. Wang L, Zhao L, Zhang X, Zhang Q, Jia Y, Wang G, Li S, Tian D, Li WH, Yang S: Large-scale identification and functional analysis of NLR genes in blast resistance in the Tetep rice genome sequence. Proc Natl Acad Sci U S A. 2019;116(37):18479–487.
28. Van de Weyer AL, Monteiro F, Furzer OJ, Nishimura MT, Cevik V, Witek K, Jones JDG, Dangl JL, Weigel D, Bemm F. A species-wide inventory of NLR genes and alleles in *Arabidopsis thaliana*. Cell. 2019;178(5):1260–72.e1214.
29. Huang Z, Qiao F, Yang B, Liu J, Liu Y, Wulff BBH, Hu P, Lv Z, Zhang R, Chen P et al. Genome-wide identification of the NLR gene family in Haynaldia villosa by SMRT-RenSeq. BMC Genomics. 2022;23(1):118.
30. Stam R, Scheikl D, Tellier A: Pooled enrichment sequencing identifies diversity and evolutionary pressures at NLR resistance genes within a wild tomato population. Genome Biol Evol. 2016;8(5):1501–1515.
31. Jupe F, Chen X, Verweij W, Witek K, Jones JD, Hein I. Genomic DNA library preparation for resistance gene enrichment and sequencing (RenSeq) in plants. Methods Mol Biol. 2014;1127:291–303.
32. Disch EM, Tong M, Kotur T, Koch G, Wolf CA, Li X, Hoth S. Membrane-associated ubiquitin ligase SAUL1 suppresses temperature- and humidity-dependent autoimmunity in Arabidopsis. Mol Plant Microbe Interact. 2016;29(1):69–80.
33. Li Z, Liu H, Ding Z, Yan J, Yu H, Pan R, Hu J, Guan Y, Hua J. Low temperature enhances plant immunity via salicylic acid pathway genes that are repressed by ethylene. Plant Physiol. 2020;182(1):626–39.
34. Zhang Y, Goritschnig S, Dong X, Li X. A gain-of-function mutation in a plant disease resistance gene leads to constitutive activation of downstream signal transduction pathways in suppressor of npr1-1, constitutive 1. Plant Cell. 2003;15(11):2636–46.
35. Koyama T: A hidden link between leaf development and senescence. Plant Sci. 2018, 276:105–10.
36. Jing HC, Schippers JH, Hille J, Dijkwel PP: Ethylene-induced leaf senescence depends on age-related changes and OLD genes in Arabidopsis. J Exp Bot. 2005, 56(421):2915–23.
37. Jing HC, Dijkwel PP: CPR5: A Jack of all trades in plants. Plant Signal Behav. 2008;3(8):562–63.
38. Jing HC, Hebeler R, Oeljeklaus S, Sitek B, Stuhler K, Meyer HE, Sturre MJ, Hille J, Warscheid B, Dijkwel PP. Early leaf senescence is associated with an altered cellular redox balance in Arabidopsis cpr5/old1 mutants. Plant Biol (Stuttg). 2008;10(Suppl 1):85–98.
39. Schippers JH, Nunes-Nesi A, Apetrei R, Hille J, Fernie AR, Dijkwel PP. The Arabidopsis onset of leaf death5 mutation of quinolinate synthase affects nicotinamide adenine dinucleotide biosynthesis and causes early ageing. Plant Cell. 2008;20(10):2909–25.
40. Tang Y, Huang A, Gu Y. Global profiling of plant nuclear membrane proteome in Arabidopsis. Nat Plants. 2020;6(7):838–47.
41. Fujitomo T, Daigo Y, Matsuda K, Ueda K, Nakamura Y. Critical function for nuclear envelope protein TMEM209 in human pulmonary carcinogenesis. Cancer Res. 2012;72(16):4110–18.
42. Weber E, Engler C, Gruetzner R, Werner S, Marillonnet S. A modular cloning system for standardized assembly of multigene constructs. PLoS One. 2011;6(2):e16765.
